# Stressors in the ICU: different perceptions of patients, relatives and staff members

**DOI:** 10.1186/cc11094

**Published:** 2012-03-20

**Authors:** M Umbrello, G Mistraletti, B Cerri, E Carloni, V Mariani, A Di Carlo, F Martinetti, L D'Amato, S Miori, G Iapichino

**Affiliations:** 1 Università degli Studi di Milano, Milan, Italy; 2 AO San Paolo - Polo Universitario, Milan, Italy

## Introduction

The high-risk critically ill are exposed to significant stressors, along with difficulties in communicating them to relatives and members of the staff. The aim of this study was to compare the perception of stressors as reported by patients (P), relatives (R) and ICU staff members (S).

## Methods

A validated questionnaire [1] was used to quantitatively assess discomforts related to the ICU stay. Items were clustered into categories; higher scores refer to a higher stressfulness. The median (IQR) was calculated for each category. Twenty-eight high-risk critically ill at discharge, 55 relatives 48 hours after admission of their next of kin, and a total of 125 staff members (55 attending physicians, 40 nurses and 30 medical students/specialist trainees) were interviewed. Fifty-six of the staff members were used to keep patients consciously sedated as for local guidelines; the remaining used deeper levels of sedation. Nonparametric tests were used as needed.

## Results

All stressor categories were differently reported by the three groups analysed: environmental (S = 17 (15 to 19), R = 15 (13 to 18), P = 10 (8 to 11), *P *< 0.01), relationships (S = 23 (21 to 25), R = 20.5 (17 to 24.5), P = 14 (11 to 17), *P *< 0.01), emotional (S = 25.5 (23 to 28), R = 24 (20 to 26), P = 18 (15 to 22), *P *< 0.01), and physical (S = 35 (31 to 38), R = 33 (26.5 to 37), P = 27 (21 to 30), *P *< 0.01). Among the staff members, nurses overestimated more than attending physicians, while trainees are closer to relatives' perception (*P *= 0.03). Staff members used to conscious sedation overestimate less the impact of environmental stressors (*P *= 0.03). Years of experience (*r *= 0.24, *P *= 0.03) and age (*r *= 0.27, *P *= 0.01) are related to stressor overestimation among staff members.

## Conclusion

Members of the staff should reconsider their beliefs on patients' perception of stressors. We argue that such an overestimation may bring inappropriate administration of analgesic and sedative drugs, particularly for nurses and older members of staff. Relatives might be useful intermediaries to have a better insight of patients' perception.
